# Microbiological and Pharmacological Aspects Involved in Dentin-Pulp Complex Regeneration: A Scoping Review

**DOI:** 10.4317/jced.62918

**Published:** 2025-09-01

**Authors:** Isabela Giraldo-Badillo, Eliana Pineda-Vélez, Belfran A Carbonell-Medina, Carlos M Ardila

**Affiliations:** 1 Master’s Program in Microbiology. Genetics, Regeneration, and Cancer Research Group. School of Microbiology, University of Antioquia, UdeA, Medellín, Colombia; 2 Endodontist. Master’s in Epidemiology. Basic Studies Department, Faculty of Dentistry, University of Antioquia, UdeA. Genetics, Regeneration, and Cancer Research Group. University of Antioquia, UdeA. Medellín, Colombia; 3 Ph.D. in Biological Sciences. Basic Studies Department, Faculty of Dentistry, University of Antioquia, UdeA. Genetics, Regeneration, and Cancer Research Group. University of Antioquia, UdeA. Medellín, Colombia; 4 Department of Periodontics, Saveetha Dental College, Saveetha Institute of Medical and technology sciences, SIMATS, Saveetha. University, Chennai, Tamil Nadu, India; 5 Ph.D in Epidemiology. Postdoc. Titular Professor. Basic Studies Department, Faculty of Dentistry, Biomedical Stomatology Research Group, School of Dentistry. Universidad de Antioquia U de A, Medellín Colombia

## Abstract

**Background:**

The regeneration of the dentin-pulp complex represents a pivotal challenge in endodontics, requiring a delicate balance between microbial eradication and tissue repair. This scoping review, conducted in accordance with PRISMA-ScR guidelines, synthesizes current evidence on microbiological and pharmacological factors influencing regenerative outcomes.

**Material and Methods:**

A systematic search of PubMed, Scopus, Web of Science, and Cochrane Library identified 242 studies, with 15 meeting inclusion criteria after screening.

**Results:**

The review highlights the dominance of anaerobic biofilm-forming pathogens (Enterococcus faecalis, *Porphyromonas *gingivalis**) in periapical lesions, their virulence mechanisms (e.g., proteolytic enzymes, immune evasion), and the rising threat of antibiotic resistance driven by β-lactamases and efflux pumps. Pharmacologically, while triple/double antibiotic pastes promote dentin thickening, their cytotoxicity at high concentrations and disruption of commensal microbiota underscore the need for optimized dosing. Emerging alternatives—such as antimicrobial peptides, calcium hypochlorite, and immunomodulatory biomolecules—demonstrate superior biocompatibility and dual action against pathogens while supporting stem cell viability.

**Conclusions:**

Future directions emphasize microbiome-targeted therapies, advanced biomaterials, and personalized approaches leveraging metagenomics. This review underscores the imperative to integrate selective antimicrobial strategies with regenerative biology to advance endodontic outcomes.

** Key words:**Dentin-pulp regeneration, endodontic infections, biofilm, antimicrobial resistance, regenerative endodontics.

## Introduction

The regeneration of the dentin-pulp complex represents a pivotal challenge in dentistry, with significant clinical and therapeutic implications for preserving dental vitality. Unlike conventional endodontic treatments, which focus on removing necrotic pulp tissue and achieving a three-dimensional seal of the root canal system, regenerative strategies aim to restore biological functionality by activating cellular and molecular mechanisms that promote repair and tissue formation [1]. In this context, microbiological and pharmacological factors play a critical role in modulating the microenvironment necessary for the success of these procedures.

Pulp and periapical infections are closely associated with complex microbial communities, predominantly composed of facultative and strict anaerobic bacteria organized in highly resistant biofilm structures [2]. The ability of these microorganisms to invade root canals, establish virulence factors, and evade the host’s immune response directly influences disease progression and the success of regenerative therapies [3]. According to studies by Al-Ahmad *et al*. [4] and Siqueira *et al*. [5], the emergence of bacterial strains with antimicrobial resistance mechanisms poses a significant challenge in selecting pharmacological treatments. These treatments must not only effectively control infection but also facilitate the restoration of pulp function. This integrated approach is essential for addressing the complexities of the microbiology of endodontic infections and improving clinical outcomes in patients with apical periodontitis.

From a pharmacological perspective, the use of antimicrobial agents, modulators of the inflammatory response, and pro-regenerative biomolecules has been extensively explored in the context of pulp regeneration [6,7]. However, the indiscriminate and inappropriate use of antibiotics can lead to adverse effects, such as disruption of the oral microbiota and the selection of resistant microorganisms, thereby compromising the long-term effectiveness of treatments [1]. Consequently, there is a pressing need to explore antibiotic tools with minimal cytotoxic effects, emphasizing the importance of standardizing concentrations, ratios, and approaches to maximize antimicrobial efficacy without compromising cellular viability or the regenerative capacity of the dentin-pulp tissue.

This review aims to analyze the microbiological and pharmacological aspects involved in dentin-pulp complex regeneration, focusing on the impact of periapical infections on the feasibility of regenerative treatments and the role of pharmacological interventions in modulating the reparative process. The main limitations of current therapies will be discussed, and new strategies to optimize dentin-pulp tissue regeneration will be explored, integrating insights from microbiology, pharmacology, and regenerative biology. Through this analysis, the study seeks to contribute to the development of more effective and targeted therapeutic approaches to improve the prognosis of regenerative endodontic treatments.

## Material and Methods

This scoping review was conducted in accordance with the Preferred Reporting Items for Systematic Reviews and Meta-Analyses extension for Scoping Reviews (PRISMA-ScR) to ensure a systematic and transparent approach to synthesizing evidence [8]. The review protocol was developed to address the research question: What are the microbiological and pharmacological factors influencing dentin-pulp complex regeneration? This question guided the identification of relevant studies and the synthesis of findings to map the current evidence landscape and identify knowledge gaps.

A comprehensive literature search was performed across multiple electronic databases, including PubMed, Scopus, Web of Science, and the Cochrane Library, covering publications from January 2010 to April 2025. This timeframe was selected to capture recent advancements in regenerative endodontics while ensuring relevance to contemporary clinical practice. The search strategy combined controlled vocabulary (e.g., MeSH terms) and free-text terms related to dentin-pulp regeneration, endodontic infections, microbial biofilms, pharmacological interventions, and regenerative endodontics. Key search terms included “dentin-pulp regeneration,” “endodontic infections,” “biofilm,” “antimicrobial resistance,” “antibiotics,” “immunomodulators,” “biomolecules,” and “regenerative endodontics,” with Boolean operators (AND, OR) used to refine the search. The strategy was tailored to each database to optimize retrieval of relevant studies.

Inclusion criteria were established to focus the review on studies pertinent to the research question. Eligible studies included those investigating microbial biofilms in endodontic infections, pharmacological interventions (e.g., antibiotics, immunomodulators, biomolecules), and regenerative endodontic outcomes, encompassing *in vitro*, *in vivo*, or clinical studies. Studies were required to be published in English and peer-reviewed to ensure quality. Exclusion criteria comprised non-English studies, case reports, editorials, opinion pieces, and non-peer-reviewed articles, as these were deemed less likely to provide robust evidence for synthesis.

The study selection process involved two independent reviewers who screened titles and abstracts against the inclusion criteria using a standardized screening tool. Discrepancies were resolved through discussion or consultation with a third reviewer to ensure consensus. Full-text articles of potentially eligible studies were retrieved and assessed for final inclusion. A PRISMA flow diagram was used to document the selection process, detailing the number of studies identified, screened, included, and excluded, along with reasons for exclusion.

Data extraction was performed by two reviewers using a pre-designed charting form tailored to the review’s objectives. Extracted data included study characteristics, microbial species investigated, pharmacological interventions, and regenerative outcomes. Data on antibiotic efficacy, biofilm resistance mechanisms, and biomolecular interactions were also charted to address the microbiological and pharmacological dimensions of the research question. Any disagreements during data extraction were resolved through discussion.

Data synthesis employed a thematic analysis approach to organize findings into meaningful categories aligned with the review’s objectives. Themes included microbiological factors (e.g., microbiota composition, virulence factors, antibiotic resistance), pharmacological factors (e.g., antibiotics, immunomodulators, pro-regenerative biomolecules), microbial-pharmacological interactions, and therapeutic alternatives. A narrative synthesis was used to describe the findings, supported by Tables and Figures where appropriate to summarize key patterns and gaps in the literature. No meta-analysis was conducted, as the scoping review aimed to map the breadth of evidence rather than quantify effect sizes.

Quality appraisal of individual studies was not performed, consistent with scoping review methodology, which prioritizes mapping the evidence over critical appraisal. However, the review considered the overall robustness of the evidence base by noting study designs and methodological limitations during synthesis. Ethical approval was not required, as the review relied on publicly available published studies.

## Results

**Figure 1:**
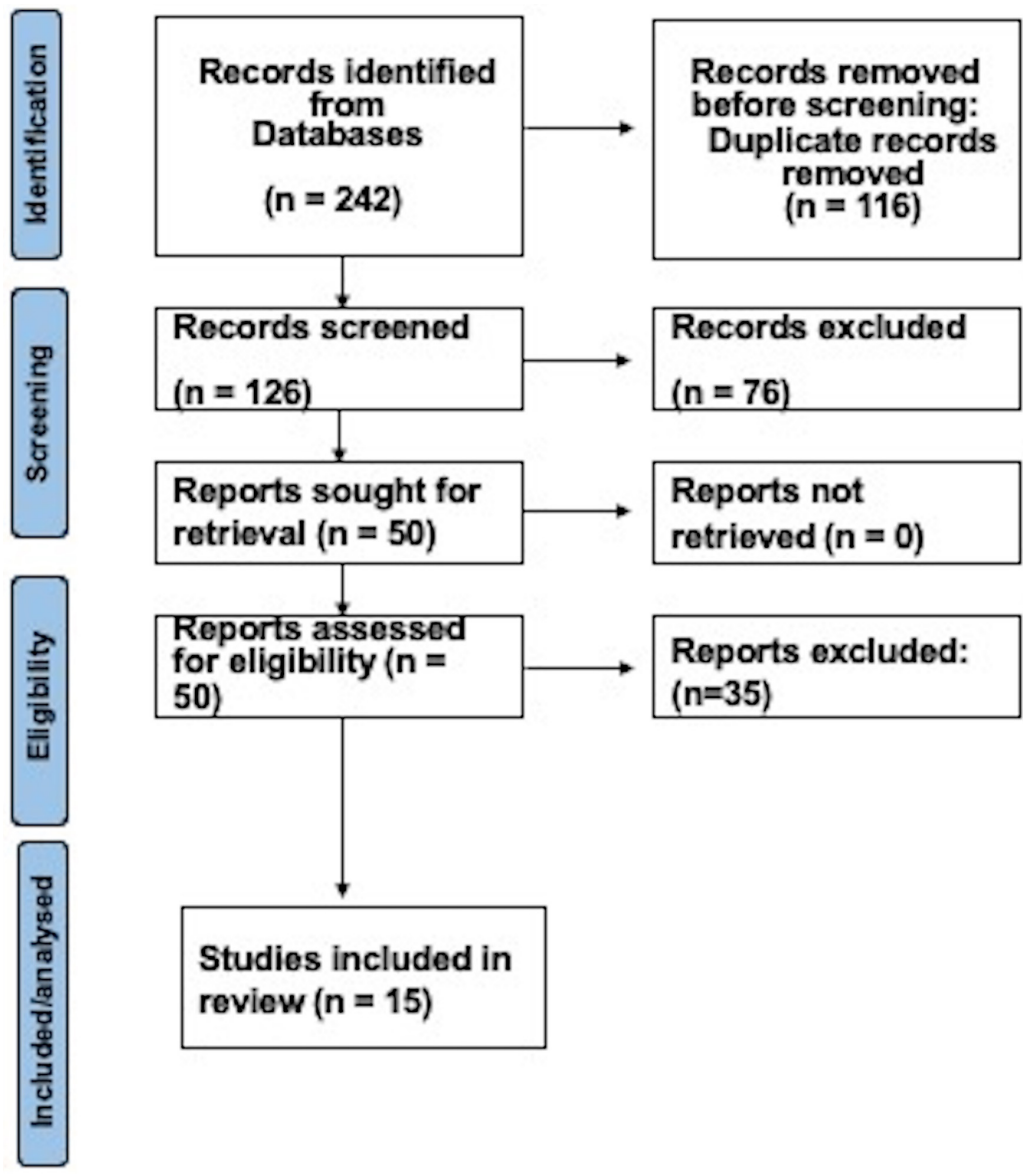
Diagram of the selection process.

A total of 242 studies were identified through the search strategy in electronic databases. Following the removal of duplicates and the application of eligibility criteria, 50 articles were selected for full-text assessment. During this assessment, studies were excluded primarily for not adequately addressing microbiological factors, pharmacological factors, microbial-pharmacological interactions, or therapeutic alternatives. The final synthesis of this scoping systematic included 15 papers (9-23). The complete search and selection process is illustrated in Figure 1.

An overview of the essential elements of the 15 studies that were part of this scoping review can be found in  Table 1. The studies comprised a mix of *in vitro* (*n*=11), clinical (*n*=1), and review (*n*=3) designs, reflecting a diverse evidence base. All studies focused on regenerative endodontics, specifically addressing microbiological and pharmacological factors influencing dentin-pulp complex regeneration. Most studies were conducted in controlled laboratory settings, clinical environments, or through narrative synthesis, with a predominant focus on teeth with pulp necrosis, periapical lesions, or experimental models of pulpal inflammation. Common methodologies included cell viability assays, microbial culture, histological and histobacteriological assessments, confocal laser scanning microscopy (CLSM), single-cell RNA sequencing, polymerase chain reaction (PCR) analysis, and meta-analytical synthesis, providing a comprehensive view of the challenges and opportunities in regenerative endodontics.

The review systematically examines key aspects influencing regenerative endodontics, beginning with microbiological factors, which detail the polymicrobial nature of periapical lesions, emphasizing biofilm-forming pathogens like *E. faecalis* and virulence mechanisms that exacerbate infections. Antibiotic Resistance Mechanisms highlight challenges posed by biofilm resilience and multidrug resistance, underscoring the limitations of conventional disinfectants. The pharmacological Factors section evaluates antibiotic pastes (TAP/DAP) and immunomodulators, noting their dual role in microbial control and unintended cytotoxic or inflammatory effects on dental pulp stem cells (DPSCs). Microbial-Pharmacological Interactions explore how broad-spectrum antibiotics disrupt symbiotic microbiota, exacerbating dysbiosis and impairing regeneration, while therapeutic alternatives propose innovative solutions like antimicrobial peptides (AMPs), probiotics, and bioactive biomaterials to selectively target pathogens while preserving tissue-healing microenvironments. Together, these sections illustrate the intricate balance required between effective antimicrobial strategies and regenerative compatibility in endodontic therapy.

Microbiological Factors

Microbiota Associated with Periapical Lesions

Periapical lesions, chronic inflammatory responses to microbial invasion of the root canal system, are driven by polymicrobial communities dominated by facultative and strict anaerobic bacteria, such as *Tannerella forsythia*, Bacteroides, *Porphyromonas *gingivalis**, *Prevotella intermedia*, *Prevotella nigrescens*, *Fusobacterium nucleatum*, *Streptococcus constellatus*, *Treponema denticola*, *Enterococcus faecalis*, Eubacterium, and *Campylobacter* species. These bacteria form highly structured biofilms that thrive in the anaerobic root canal environment, evading conventional endodontic treatments and host immune responses [9]. *E. faecalis*, in particular, contributes to persistent infections due to its robust biofilm-forming capacity. Infections were classified as primary, secondary, or persistent, with primary infections featuring strict anaerobes and secondary/persistent infections showing microbiota shifts due to antimicrobial selective pressure [10]. Fungi like *Candida albicans* were also implicated in endodontic failures, with biofilms conferring 10–100 times greater resistance to treatments. Advanced metagenomic sequencing revealed 177 genera and 340 species in endodontic infections, highlighting the complexity of these microbial communities and the need for precise therapeutic targeting [9,10].

Virulence and Pathogenicity Factors

Microbial virulence factors significantly drive the progression of endodontic infections. Proteolytic enzymes, such as gingipains from *P. *gingivalis**, degrade host proteins, facilitating tissue invasion and immune evasion [11]. Lipopolysaccharides and exotoxins from species like *P. gingivalis* and *P. intermedia* trigger intense inflammatory responses, contributing to pulp and periapical tissue destruction. Biofilm formation, particularly by *E. faecalis*, confers up to 1000-fold resistance to antibiotics, complicating eradication. *F. nucleatum* acts as an ecological bridge, enhancing biofilm stability through co-adhesion. Treponema species, notably T. denticola, modulate host immune responses by reducing neutrophil phagocytosis, promoting chronic inflammation. Residual bacteria post-treatment were shown to cause localized dentin demineralization, underscoring the challenge of complete microbial elimination [12].

Antibiotic Resistance Mechanisms in Pulp Infections

Antibiotic resistance poses a major barrier to regenerative endodontics, driven by mechanisms such as β-lactamase production, target site modification, and efflux pump activation [13,14]. Biofilms act as physical and biochemical barriers, reducing antimicrobial penetration and efficacy. Frequent antibiotic use has led to multidrug-resistant strains, complicating chronic infection resolution. Standard disinfection protocols, including sodium hypochlorite (NaOCl), EDTA, and calcium hydroxide Ca(OH)2, reduce microbial load but often fail to eliminate resistant bacteria. Studies exploring double antibiotic pastes (DAP) found that moderate concentrations preserve dental pulp stem cell (DPSC) proliferation, while high concentrations induce cytotoxicity [15,16]. Antibiotic-eluting nanofibers and calcium hypochlorite showed promise in reducing biofilms with lower cytotoxicity, supporting their potential in regenerative therapies [17,18].

Pharmacological Factors

Antibiotics in Regenerative Therapy

Antibiotics are central to endodontic regenerative therapy (RET), particularly in immature teeth with pulp necrosis, where they facilitate microbial elimination to create a regenerative microenvironment. Triple antibiotic paste (TAP) and double antibiotic paste (DAP) are commonly used, with DAP avoiding minocycline to prevent discoloration. In vitro studies showed DAP at moderate concentrations does not impair DPSC viability but reduces mineralization, affecting long-term regenerative potential [15]. Clinical studies reported DAP and calcium hydroxide promote dentin wall thickening, with DAP showing superior structural outcomes [19,20]. A meta-analysis of 32 studies confirmed TAP/DAP’s association with dentin thickening, though root length gains were comparable to calcium hydroxide [21]. Calcium hypochlorite outperformed NaOCl in antimicrobial efficacy and dentin preservation, supporting its use in RET [17,18]. High antibiotic concentrations were cytotoxic, reducing DPSC proliferation and odontogenic differentiation, necessitating dose optimization [19,20].

Modulators of the Immune Response

Immune modulation is critical for successful dentin regeneration, as chronic inflammation can hinder tissue repair. Tertiary lymphoid structures (TLS) in chronic infections were found to organize immune responses, potentially aiding regeneration [22]. High concentrations of TAP/DAP increased proinflammatory cytokines, perpetuating inflammation and impairing DPSC function [15,16]. Anti-inflammatory agents like TGF-β and interleukins were proposed to restore immune homeostasis, enhancing regeneration [21]. Biomaterials releasing immunoregulatory agents and inhibiting NF-κB pathways reduced inflammation and supported cell proliferation [20,21]. Clinical studies combining antimicrobial and immunomodulatory strategies showed improved outcomes by preserving periapical tissue responses [19,20].

Pro-Regenerative Biomolecules

Antimicrobial peptides (AMPs) emerged as dual-action agents, combating resistant bacteria while promoting DPSC proliferation and differentiation [22]. Unlike TAP/DAP, AMPs preserved cell viability and supported matrix remodeling, creating a regenerative microenvironment [15]. Growth factors like PDGF and bFGF enhanced angiogenesis and dentin formation, with scaffold-based delivery improving outcomes [17,19-20). Matrix metalloproteinase (MMP) inhibitors reduced collagen degradation, aiding tissue organization [21]. Calcium hypochlorite preserved dentin matrix proteins, supporting cell anchorage [18]. High antibiotic concentrations inhibited differentiation genes, highlighting the need for biomolecule integration to counter these effects [16].

Microbial and Pharmacological Interactions

Broad-spectrum antibiotics, such as TAP, disrupt oral microbiota, causing dysbiosis and favoring resistant pathogens, which compromises regenerative outcomes. Commensal bacteria like *Streptococcus sanguinis* and *Veillonella* spp. regulate immune responses, but their reduction by antibiotics increases proinflammatory cytokines (IL-6, TNF-α), hindering DPSC differentiation. High TAP concentrations induced DPSC cytotoxicity via metabolic disruption and oxidative stress, limiting regenerative potential. Antibiotics also altered MMP-2 and MMP-9 activity, affecting matrix remodeling and growth factor release. Alternatives like AMPs, nanoparticles, and probiotics showed promise in preserving microbiota and supporting regeneration, emphasizing the need for selective antimicrobial strategies [15,16,21].

Therapeutic Alternatives

Emerging therapies aim to balance pathogen elimination with microbiota preservation. AMPs (e.g., LL-37, β-defensin-3) targeted resistant pathogens like *Enterococcus faecalis* while promoting regeneration, with low resistance risk [23]. Probiotics (Lactobacillus reuteri, *Streptococcus* salivarius) and prebiotics reduced pathogenic load and inflammation, supporting microbial diversity. Bioactive biomaterials, such as silver/copper-doped bioceramics and polylactic acid, provided controlled antimicrobial activity without compromising cell viability, offering biocompatible disinfection options. These alternatives represent a shift toward selective, regenerative-focused endodontic therapies [15,16,21].

## Discussion

The findings of this scoping review highlight the intricate interplay between microbiological and pharmacological factors in dentin-pulp complex regeneration, revealing both advancements and persistent challenges. The dominance of anaerobic bacteria, particularly *E. faecalis* and *P. *gingivalis**, in periapical lesions underscores the difficulty of eradicating biofilms, which confer significant resistance to conventional treatments [5,3]. The identification of 177 genera and 340 species through metagenomic sequencing emphasizes the need for precise microbial characterization to inform targeted therapies [10]. Virulence factors, such as gingipains and immune-modulating mechanisms, further complicate infection control, as seen with T. denticola’s ability to reduce phagocytosis [24,25]. Antibiotic resistance, driven by biofilms and mechanisms like β-lactamases, limits the efficacy of standard protocols, necessitating innovative approaches [13,26].

Pharmacologically, TAP and DAP demonstrate regenerative potential by promoting dentin thickening, but their cytotoxicity at high concentrations highlights the need for dose optimization [15,16,21]. Calcium hypochlorite’s superior biocompatibility compared to NaOCl suggests a shift toward less aggressive irrigants to preserve dentin integrity [17,18]. The immunomodulatory role of antibiotics, potentially influencing TLS formation, indicates a broader impact on tissue repair, though this requires further exploration [22]. Pro-regenerative biomolecules, such as AMPs and growth factors, offer promising alternatives by balancing antimicrobial action with tissue regeneration, contrasting with the limitations of traditional antibiotics [19,22]. The adverse effects of antibiotics on oral microbiota, including dysbiosis and increased inflammation, underscore the need for selective therapies like probiotics and biomaterials that preserve commensal bacteria [23].

The review’s findings align with the need for new therapeutic approaches in endodontics, as prolonged antibiotic use exacerbates resistance and disrupts the pulp microenvironment [27]. Combined therapies integrating AMPs, MMP inhibitors, and nanoparticles enhance pathogen elimination while maintaining tissue homeostasis, offering a synergistic approach to infection management (28,29]. Targeted strategies, such as bacteriophages and photodynamic therapy, provide selective antimicrobial action, minimizing collateral damage to beneficial microbiota [29,30]. These advancements necessitate a restructuring of clinical protocols, incorporating biomarkers and metagenomic tools for personalized therapy. The development of advanced experimental models, like dentin-pulp organoids, could further refine preclinical validation, bridging the gap between research and clinical application [31,32].

This scoping review has several limitations. The exclusion of non-English studies may have omitted relevant findings from diverse contexts. The heterogeneity of study designs (*in vitro*, *in vivo*, clinical) and outcomes limited direct comparisons, reflecting the broad scope of regenerative endodontics. The absence of quality appraisal, typical for scoping reviews, means methodological rigor was not formally assessed, potentially influencing the reliability of some findings. The reliance on studies from 2010–2025 may have missed earlier foundational work, though this was intentional to focus on recent advancements. Finally, the limited number of clinical studies compared to *in vitro* and *in vivo* studies suggests a gap in translating laboratory findings to clinical practice [33].

Future research should prioritize long-term clinical trials to validate the efficacy and safety of emerging therapies like AMPs, probiotics, and bioactive biomaterials. Studies should focus on optimizing antibiotic concentrations and delivery systems to minimize cytotoxicity while maximizing regenerative outcomes. The integration of metagenomic sequencing and biomarkers in clinical practice could enable personalized endodontic therapies, tailoring interventions to individual microbial profiles. Multidisciplinary research combining microbiology, biotechnology, and biomaterial engineering is essential to develop innovative models, such as organoids, for testing new treatments. Addressing economic and regulatory barriers will be critical to ensure the accessibility of these therapies. Finally, guidelines incorporating selective antimicrobial strategies and immunomodulatory approaches should be developed to standardize regenerative endodontic protocols.

## Conclusions

This scoping review provides a comprehensive overview of the microbiological and pharmacological factors influencing dentin-pulp complex regeneration. Anaerobic bacteria and biofilms, coupled with antibiotic resistance, pose significant challenges to regenerative endodontics, necessitating targeted and biocompatible therapies. While TAP and DAP support dentin thickening, their cytotoxicity at high concentrations highlights the need for dose optimization and alternative agents like calcium hypochlorite and AMPs. Pro-regenerative biomolecules and immunomodulatory strategies offer promising avenues for enhancing tissue repair while controlling infection. The adverse effects of antibiotics on oral microbiota underscore the importance of selective therapies, such as probiotics and bioactive biomaterials, to preserve microbial homeostasis. Combined and targeted approaches, supported by advanced diagnostics, hold the potential to revolutionize regenerative endodontics, improving clinical outcomes. Continued research and clinical translation are essential to overcome current limitations and realize the full potential of these innovative therapies.

## Figures and Tables

**Table 1:** Summary of Included Studies.

Study Design	Population/Sample	Intervention or Exposure	Variables Evaluated	Key Findings	Conclusions	Reference
In vitro	Human dental pulp stem cells (DPSCs) were used to assess responses to different antibiotic paste formulations.	Double antibiotic pastes (DAP) composed of metronidazole and ciprofloxacin (1:1) with radiopaque agents (barium sulfate [BaSO] and zirconium oxide [ZrO]) at concentrations of 1 mg/mL and 5 mg/mL.	DPSC proliferation after 3 days. Alkaline phosphatase (ALP) activity and mineral deposition after 7 and 14 days.	No significant differences in DPSC proliferation between DAP and controls (p < 0.05). DAP reduced mineral nodule formation, more pronounced at higher antibiotic concentrations. Radiopaque agents alone had no negative effects on mineral deposition or proliferation.	Authors conclude that DAP with BaSO or ZrO up to 5 mg/mL is not cytotoxic and does not inhibit DPSC proliferation, but antibiotics reduce mineral nodule formation dose-dependently. Low effective concentrations are recommended for optimal biocompatibility and antimicrobial activity.	15
Prospective study	22 teeth with necrotic immature roots from patients with symptomatic immature permanent teeth, no antibiotic allergies, and informed consent.	Teeth treated with: Calcium hydroxide (conventional). Diluted MC-TAP gel (1 mg TAP). Diluted MC-DAP gel (1 mg DAP).	DPSC proliferation and differentiation (WST and ALP assays). Clinical sign/symptom resolution. Dental vitality at 12-24-month follow-up.	All treatments resolved symptoms short-term. MC-DAP showed symptom recurrence long-term. All teeth were symptom-free at 12-24 months, but no vitality was achieved. High antibiotic concentrations (500-1000 mg/mL) were toxic to DPSCs; diluted concentrations (0.1-2 mg/mL) were not.	Calcium hydroxide and MC-TAP were clinically successful, but MC-DAP use is discouraged due to poor regeneration outcomes.	19
Meta-analysis	32 original articles, totaling 758 regenerative endodontic procedures on immature permanent teeth with pulpal necrosis or periapical pathology.	Evaluated two main treatments: antibiotic pastes (mostly modified) and calcium hydroxide (Ca(OH) during disinfection.	Dentin wall thickening, apical closure, apical repair, root lengthening.	Dentin wall thickening in 66% of antibiotic paste cases vs. 53% with Ca(OH). Apical closure in 66% with antibiotics vs. 88% with Ca(OH). Similar apical repair and root lengthening outcomes with differences in effectiveness.	Significant differences favor antibiotic pastes for dentin thickening and Ca(OH) for apical closure. Choice should depend on root development stage to improve predictability and reduce fracture risk.	21
In vitro	215 extracted human single-rooted teeth with complete root development, no morphological anomalies, stored at -20°C.	Teeth infected with Enterococcus faecalis and treated with sodium hypochlorite (NaOCl) and calcium hypochlorite Ca(OCl) at 0.5%, 2.5%, and 6% for 15, 30, and 60 seconds.	Antimicrobial efficacy, bacterial viability in dentinal tubules (CLSM), dentin organic matrix damage (picrosirius staining and optical microscopy).	Both NaOCl and Ca(OCl) showed similar antimicrobial activity, but Ca(OCl) caused less collagen fiber damage, suggesting lower cytotoxicity.	Both solutions have comparable antimicrobial efficacy, but Ca(OCl) offers less collagen damage. Clinical trials are needed to validate findings.	18
In vitro	30 root canal samples infected with a 3-week Enterococcus faecalis biofilm, divided into three groups (10 each) for TAP, Ca(OH), and Ca(OCl), with positive and negative controls.	Root canals treated for 7 days with TAP, Ca(OH), or Ca(OCl); 10% ascorbic acid used to neutralize Ca(OCl) effects before cell seeding.	Colony-forming unit (CFU) reduction, cell viability, ALP activity, morphological analysis of dental pulp stem cells.	Ca(OCl) was most effective in reducing bacterial load, but reduced cell viability and ALP activity. 10% ascorbic acid neutralized toxicity, restoring viability and ALP to control levels.	Ca(OCl) is effective for disinfection but toxic to cells; ascorbic acid mitigates this, supporting its use in regenerative endodontics with balanced antimicrobial and biocompatible outcomes.	17
In vitro	Fibrin hydrogels and self-assembling peptides (SAP) prepared in the lab, with human dental pulp stem cells (hDPSCs).	Incorporation of COAM into hydrogel compositions, assessed for microstructural and mechanical properties (homogeneity, fiber aggregation, elasticity, cell viability).	Hydrogel microstructure (roughness, fiber length, diameter, straightness, alignment). Mechanical stability (elastic modulus). hDPSC viability, shape, proliferation, DNA content.	COAM did not alter fibrin hydrogel microstructure but caused aggregation in SAP hydrogels. SAP hydrogels were 7x stiffer than fibrin hydrogels. hDPSC viability and adhesion were higher in fibrin hydrogels. Higher DNA content in fibrin hydrogels.	Fibrin hydrogels with COAM show microstructural stability and better hDPSC viability/DNA content than SAP, promising for endodontic regeneration; further research on COAM interactions is needed.	23
In vitro	Human dentin discs from extracted teeth, prepared to simulate immature roots with open apices.	Effect of triple antibiotic paste (TAP), double antibiotic paste (DAP), and calcium hydroxide Ca(OH) on apical papilla stem cell (SCAP) viability at 1 mg/mL and 1000 mg/mL for 7 and 28 days.	SCAP viability (CellTiter-Glo luminescence assay), survival, and proliferation.	TAP/DAP at 1000 mg/mL eliminated SCAP viability; 1 mg/mL had no effect. Ca(OH) significantly increased SCAP survival/proliferation vs. control.	TAP/DAP at common concentrations impair SCAP survival, while Ca(OH) promotes it. Careful selection of medications and concentrations is critical.	16
In vitro	Human dental pulp stem cells (DPSCs) and Enterococcus faecalis (ATCC 29212) for cytotoxicity testing.	Dilutions of triple antibiotic paste (TAP) and double antibiotic paste (DAP) at 0.125, 0.25, 0.5, 1, and 10 mg/mL exposed to a 3-day E. faecalis biofilm.	Antibacterial effects (CFU/mL reduction). Cytotoxicity (LDH and WST-1 assays on DPSCs).	All dilutions reduced E. faecalis CFU/mL. Only 0.125 mg/mL TAP/DAP spared DPSC viability; higher concentrations caused significant cytotoxicity.	Adequate antibiotic dilutions are essential for disinfection without compromising DPSC viability, suggesting low concentrations for clinical relevance in regeneration.	20
In vitro	Healthy and inflamed human dental pulp samples, plus murine pulpitis models, including immune cells (T cells, B cells, macrophages).	Assessed immune response to bacterial infection, focusing on tertiary lymphoid structure (TLS) formation in inflammation.	Pulp cellular composition, TLS markers (CCL19, LAMP3, CCR7, CD86), immune cell communication in pulpitis.	Inflamed pulp showed increased immune cell types vs. healthy pulp; TLS improved immune response, with CCL3 as a key TLS driver.	TLS in dental pulp is crucial for bacterial defense, enhancing immune cell infiltration and communication, highlighting its immunological role.	22
In vitro	15 upper incisors with pulpal necrosis and immature roots (7-17 years), trauma-related lesions, randomized to triple antibiotic paste or Ca(OH) with chlorhexidine.	Regenerative endodontic procedures with 6% NaOCl irrigation and inter-appointment medication (triple antibiotic paste or Ca(OH) with chlorhexidine); samples taken pre- and post-treatment.	Bacterial levels pre-treatment (S1), post-irrigation (S2), post-medication (S3). Clinical/radiographic outcomes, dentin wall thickness, apical closure.	All teeth had bacteria pre-treatment; irrigation reduced positives, but residual bacteria negatively impacted dentin wall thickness. Complete apical healing was achieved despite residuals.	Inter-appointment medication enhances disinfection in necrotic immature teeth; both treatments were similarly effective, with residual bacteria affecting healing. Larger studies are needed.	12
In vitro	24 canine teeth from 6 male 70-day-old subjects with induced periapical lesions.	Compared traditional revascularization (blood clot scaffold) with pulp regeneration (hydrogel-encapsulated dental pulp stem cells).	Radiographic root development, root wall thickness, periapical radiolucency, histological/histobacteriological findings.	No significant radiographic differences between groups (P > 0.05). Residual bacteria correlated with reduced growth and mineralization (P < 0.001).	Residual bacteria critically impair regenerative outcomes; effective disinfection protocols are essential for success.	13
Review	Clinical case series on immature teeth with pulpal necrosis and affected dental tissue, focusing on regenerative procedure outcomes.	Evaluated regenerative endodontics in immature teeth with/without prior pulpal infection, using antimicrobial strategies and regeneration techniques.	Infection control, antimicrobial strategy effectiveness, revascularization degree, root thickness/length, stem cell response.	Preoperative infection significantly predicts outcomes. Antimicrobial strategies need to be more thorough, preserving dentin bioactivity.	Regenerative endodontics has potential for necrotic immature teeth, but effective infection management and tailored disinfection protocols are critical.	14
In vitro	Mouse calvarial osteoblastic precursors (C57BL/6 newborns).	Osteoblastic precursors exposed to heat-killed E. faecalis (HKEF) at 1 x 10, 1 x 10, 1 x 10, or 1 x 10 CFU/mL for 48 hours with ascorbic acid and -glycerophosphate.	Mineralization (% via Alizarin Red S staining). Osteogenic gene expression (Runx2, osterix, -catenin, osteocalcin, collagen I) by PCR. Chemokine secretion (KC, MCP-1) by ELISA. PBMC migration (trans well assay).	HKEF inhibited mineralization and Runx2 expression, increased KC/MCP-1, suggesting a role in inflammation and bone loss in refractory apical periodontitis.	E. faecalis inhibits osteoblastic differentiation and promotes inflammation, complicating bone regeneration in periodontitis.	10
Review	Compilation of existing studies on pathogen recognition receptors (PRRs) in innate immunity of dental pulp.	Assessed PRR expression (e.g., Toll-like receptors [TLRs]) in dental pulp/periapical tissues, activation mechanisms, and innate immune response.	PRR expression/function, TLR interactions with pathogen-associated molecular patterns (PAMPs), inflammatory response, innate immunity activation.	PRRs initiate innate immunity in pulp; odontoblasts express PRRs, responding to pathogens; pulp anatomy influences inflammation.	Understanding PRRs in pulp is key for immunomodulation and therapeutic targets in endodontics, potentially leading to new dental health interventions.	9
In vitro	Human dental pulp progenitor fibroblasts from extracted teeth	Pulp fibroblasts stimulated with lipopolysaccharide (LPS) from Gram-negative bacteria to assess membrane attack complex (MAC) and C5a release.	MAC formation in fibroblasts. C5a levels in culture medium. Progenitor cell migration toward LPS (transwell assay).	LPS induced MAC formation and C5a release; C5a promoted progenitor migration, inhibited by a C5aR antagonist.	Complement activation by LPS recruits pulp progenitors, suggesting implications for dentin-pulp regeneration and therapeutic signaling pathways.	11

## Data Availability

The datasets used and/or analyzed during the current study are available from the corresponding author.

## References

[1] Ribeiro JS, Münchow EA, Ferreira Bordini EA, de Oliveira da Rosa WL, Bottino MC (2020). Antimicrobial therapeutics in regenerative endodontics: A scoping review. J Endod.

[2] Hamed SA, Shabayek S, Hassan HY (2022). Biofilm elimination from infected root canals using four different single files. BMC Oral Health.

[3] Neelakantan P, Romero M, Vera J, Daood U, Khan AU, Yan A (2017). Biofilms in endodontics-Current status and future directions. Int J Mol Sci.

[4] Al-Ahmad A, Ameen H, Pelz K, Karygianni L, Wittmer A, Anderson AC (2014). Antibiotic resistance and capacity for biofilm formation of different bacteria isolated from endodontic infections associated with root-filled teeth. J Endod.

[5] Siqueira JF Jr, Rôças IN (2022). Present status and future directions: Microbiology of endodontic infections. Int Endod J.

[6] Yoshida K, Suzuki S, Kawada-Matsuo M, Nakanishi J, Hirata-Tsuchiya S, Komatsuzawa H (2019). Heparin-LL37 complexes are less cytotoxic for human dental pulp cells and have undiminished antimicrobial and LPS-neutralizing abilities. Int Endod J.

[7] Fu Z, Zhuang Y, Cui J, Sheng R, Tomás H, Rodrigues J (2022). Development and challenges of cells- and materials-based tooth regeneration. Eng Regen.

[8] Page MJ, McKenzie JE, Bossuyt PM, Boutron I, Hoffmann TC, Mulrow CD (2021). The PRISMA 2020 statement: An updated guideline for reporting systematic reviews. Int J Surg.

[9] Jang JH, Shin HW, Lee JM, Lee HW, Kim EC, Park SH (2015). An overview of pathogen recognition receptors for innate immunity in dental pulp. Mediators Inflamm.

[10] Park OJ, Kim J, Yang J, Yun CH, Han SH (2015). Enterococcus faecalis Inhibits Osteoblast Differentiation and Induces Chemokine Expression. J Endod.

[11] Chmilewsky F, Jeanneau C, Laurent P, About I (2014). Pulp fibroblasts synthesize functional complement proteins involved in initiating dentin-pulp regeneration. Am J Pathol.

[12] De-Jesus-Soares A, Prado MC, Nardello LCL, Pereira AC, Cerqueira-Neto ACCL, Nagata JY (2020). Clinical and molecular microbiological evaluation of regenerative endodontic procedures in immature permanent teeth. J Endod.

[13] Verma P, Nosrat A, Kim JR, Price JB, Wang P, Bair E (2017). Effect of Residual Bacteria on the Outcome of Pulp Regeneration In Vivo. J Dent Res.

[14] Fouad AF, Verma P (2014). Healing after regenerative procedures with and without pulpal infection. J Endod.

[15] Wu JL, McIntyre PW, Hong JM, Yassen GH, Bruzzaniti A (2020). Effects of radiopaque double antibiotic pastes on the proliferation, alkaline phosphatase activity and mineral deposition of dental pulp stem cells. Arch Oral Biol.

[16] Althumairy RI, Teixeira FB, Diogenes A (2014). Effect of dentin conditioning with intracanal medicaments on survival of stem cells of apical papilla. J Endod.

[17] Alfadda S, Alquria T, Karaismailoglu E, Aksel H, Azim AA (2021). Antibacterial effect and bioactivity of innovative and currently used intracanal medicaments in regenerative endodontics. J Endod.

[18] Lodi E, Bello YD, Duarte KBDP, Montagner F, Cecchin D (2024). Antimicrobial efficacy and dentin collagen damage caused by calcium hypochlorite and sodium hypochlorite. Braz Dent J.

[19] Sabrah AH, Hammad MM, Wahab FK, AlHadidi A, Salim NA, Alelaimat AF (2023). A prospective case series in Regenerative endodontics: the effective use of diluted antibiotic hydrogels in endodontic regeneration procedures. Saudi Dent J.

[20] Sabrah AH, Yassen GH, Liu WC, Goebel WS, Gregory RL, Platt JA (2015). The effect of diluted triple and double antibiotic pastes on dental pulp stem cells and established Enterococcus faecalis biofilm. Clin Oral Investig.

[21] Báez V, Corcos L, Morgillo F, Imperatrice L, Gualtieri AF (2022). Meta-analysis of regenerative endodontics outcomes with antibiotics pastes and calcium hydroxide. The apex of the iceberg. J Oral Biol Craniofac Res.

[22] Li XL, Fan W, Fan B (2024). Dental pulp regeneration strategies: A review of status quo and recent advances. Bioact Mater.

[23] EzEldeen M, Toprakhisar B, Murgia D, Smisdom N, Deschaume O, Bartic C (2021). Chlorite oxidized oxyamylose differentially influences the microstructure of fibrin and self-assembling peptide hydrogels as well as dental pulp stem cell behavior. Sci Rep.

[24] Leite FRM, Nascimento GG, Demarco FF, Gomes BPFA, Pucci CR, Martinho FC (2015). Prevalence of Treponema species detected in endodontic infections: systematic review and meta-regression analysis. J Endod.

[25] Feng Y, Liu M, Liu Y, Li H (2024). Invasion of human dental pulp fibroblasts by Porphyromonas gingivalis leads to autophagy via the phosphoinositide 3-kinase/Akt/mammalian target of rapamycin signaling pathway. J Oral Biosci.

[26] Chrisostomo DA, Pereira JA, Scaffa PMC, Gouveia Z, Abuna GF, Plotnikov SV (2025). Antibiofilm properties, cytotoxicity, and effect on protease activity of antibiotics and EGCG-based medications for endodontic purposes. J Dent.

[27] Elgun T, Merdan YE (2023). Effect of Motiflor AS probiotic for oral health on cell viability in human gingival fibroblasts and human dental pulp stem cells. J Conserv Dent Endod.

[28] Boreak N, Alrajab EA, Nahari RA, Najmi LE, Masmali MA, Ghawi AA (2024). Unveiling therapeutic potential: targeting Fusobacterium nucleatum’s lipopolysaccharide biosynthesis for endodontic infections-an in silico screening study. Int J Mol Sci.

[29] Park OJ, Yi H, Jeon JH, Cho EB, Kum KY, Han SH (2024). Microbiota association and profiling of gingival sulci and root canals of teeth with primary or secondary/persistent endodontic infections. J Endod.

[30] Ardila CM, Bedoya-García JA (2022). Antimicrobial resistance in patients with odontogenic infections: A systematic scoping review of prospective and experimental studies. J Clin Exp Dent.

[31] Ardila CM, Jiménez-Arbeláez GA, Vivares-Builes AM (2023). Potential Clinical Application of Organs-on-a-Chip in Periodontal Diseases: A Systematic Review of In Vitro Studies. Dent J (Basel).

[32] Ardila CM, Zuluaga-Gómez M, Vivares-Builes AM (2023). Applications of Lab on a Chip in Antimicrobial Susceptibility of Staphylococcus aureus: A Systematic Review. Medicina (Kaunas).

[33] Ardila CM, González-Arroyave D, Tobón S (2025). Machine learning for predicting antimicrobial resistance in critical and high-priority pathogens: A systematic review considering antimicrobial susceptibility tests in real-world healthcare settings. PLoS One.

